# Microbially induced calcite precipitation performance of multiple landfill indigenous bacteria compared to a commercially available bacteria in porous media

**DOI:** 10.1371/journal.pone.0254676

**Published:** 2021-07-16

**Authors:** Adharsh Rajasekar, Charles K. S. Moy, Stephen Wilkinson, Raju Sekar

**Affiliations:** 1 Jiangsu Key Laboratory of Atmospheric Environment Monitoring and Pollution Control (AEMPC), Collaborative Innovation Center of Atmospheric Environment and Equipment Technology (CIC-AEET), Nanjing University of Information Science &Technology, Nanjing, China; 2 Department of Civil Engineering, Xi’an Jiaotong-Liverpool University, Suzhou, Jiangsu, China; 3 Faculty of Engineering and Information Sciences, University of Wollongong in Dubai, Dubai, UAE; 4 Department of Biological Sciences, Xi’an Jiaotong-Liverpool University, Suzhou, Jiangsu, China; Beijing Normal University, CHINA

## Abstract

Microbially Induced Carbonate Precipitation (MICP) is currently viewed as one of the potential prominent processes for field applications towards the prevention of soil erosion, healing cracks in bricks, and groundwater contamination. Typically, the bacteria involved in MICP manipulate their environment leading to calcite precipitation with an enzyme such as urease, causing calcite crystals to form on the surface of grains forming cementation bonds between particles that help in reducing soil permeability and increase overall compressive strength. In this paper, the main focus is to study the MICP performance of three indigenous landfill bacteria against a well-known commercially bought MICP bacteria (*Bacillus megaterium*) using sand columns. In order to check the viability of the method for potential field conditions, the tests were carried out at slightly less favourable environmental conditions, i.e., at temperatures between 15-17°C and without the addition of urease enzymes. Furthermore, the sand was loose without any compaction to imitate real ground conditions. The results showed that the indigenous bacteria yielded similar permeability reduction (4.79 E-05 to 5.65 E-05) and calcium carbonate formation (14.4–14.7%) to the control bacteria (*Bacillus megaterium*), which had permeability reduction of 4.56 E-5 and CaCO_3_ of 13.6%. Also, reasonably good unconfined compressive strengths (160–258 kPa) were noted for the indigenous bacteria samples (160 kPa). SEM and XRD showed the variation of biocrystals formation mainly detected as Calcite and Vaterite. Overall, all of the indigenous bacteria performed slightly better than the control bacteria in strength, permeability, and CaCO_3_ precipitation. In retrospect, this study provides clear evidence that the indigenous bacteria in such environments can provide similar calcite precipitation potential as well-documented bacteria from cell culture banks. Hence, the idea of MICP field application through biostimulation of indigenous bacteria rather than bioaugmentation can become a reality in the near future.

## Introduction

Applied geomicrobial engineering is an expanding field of studies, including the utilisation of microorganisms to modify the soil chemistry and physical properties for geotechnical purposes [1–12]. One promising form of biomineralisation for engineering applications is Microbially Induced Carbonate Precipitation (MICP) [13,14], the MICP process involves the formation and precipitation of CaCO_3_ polymorphs (e.g. calcite, vaterite, etc) as a result of changes in the soil environment (mainly pH and increasing the concentration of aqueous CO_2_) induced by microorganisms’ activities [15]. Microbial metabolic activities associated with MICP include ureolysis, denitrification, ammonification, sulphate reduction, and methane oxidation [4]. Different microorganisms have been found capable of MICP and the most studied urease bacteria are *Sporosarcina pasteurii [6,16–21] and Bacillus [22–25].* A recent review paper compared the performances of those microorganism towards MICP [26]. This technique has the potential for several geoengineering applications since microbes are small, pervasive, and they can enter into the interstices of geological materials, such as masonry or fine-grained soils, thus allowing CaCO_3_ precipitation within the materials’ granular matrix.

MICP can lead to biocementation which is the generation of bonds (cements) between soil particles caused by Ca^2+^-based mineral polymorphs [27–29]. Such bonds act as bridges between particles resulting in an increase in the shear strength [30–34] and a reduction of the void space between particles (and therefore a reduction in the material permeability) [2,35,36]. Numerous studies shown above have indicated the potential and possibility of using MICP to improve the sand strength with a minor problem being the movement of treatment depth since treating potential wastelands with larger depth might be a constraint [37]. Also, depth is often associated with a major reduction in permeability [38]. The bacteria and the cementation reagent are mixed before injected into sand columns under atmospheric or high pressure (depending on depth) to avoid clogging of the system near the injection point. For the purposes of soil improvement, the reduction of permeability is often viewed as undesirable because this may affect natural groundwater flow paths by increasing pore pressure in the soil. Hence, retaining permeability while providing strengthening treatments is ideal. MICP has proven to be a suitable technique for such situations allowing strengthening with minimum disturbances to the ground/soil conditions [39,40].

Experiments that have assessed the performance of MICP microorganisms are often conducted at temperatures ranging from 20°C to 50°C [22], elevated pressures up to 30kPa [40], and under high initial pH [21]. Limited assessments have been made to test the potential of MICP/biocementation under less favourable conditions such as lower temperatures. Similarly, study of indigenous microorganisms and their potential application for MICP/biocementation are rather limited to certain environments [2,3,6,29,41]. For example, Kang et al. (2015) [42] investigated the bioremediation potential of lead from ureolytic bacteria from abandoned metal mines in Korea. Similarly, a study by Zamarreňo et al. (2009) [43] looked at the biocalcification performance of non-sporing freshwater bacteria. There are no known references focussing on the performance of indigenous bacteria in landfills. Therefore, the approach shown in this paper is based towards assessing the biocementation potential of microorganisms isolated from landfill groundwater and leachate under realistic soil conditions. Our objective is to evaluate the MICP/biocementation potential of bacteria strains indigenous to environments where MICP might be applied including polluted habitats and areas of rapid urbanization (conversion from an agricultural use). This would reduce the costs associated to the technique and mitigate environmental risks of introducing non-indigenous species into the environment.

## Materials and methods

### Identification of bacteria and their precipitation potential

The three bacteria mentioned in this paper were isolated from landfill leachate (L3 and L5) and groundwater (W1) located in Suzhou, Jiangsu, China. The pH of the leachate and groundwater samples was alkaline in nature. The description of the landfill is mentioned in detail by [44].

A culture-dependent technique was used for bacteria isolation from the landfill leachate and groundwater samples. The isolation protocol and the sequencing methodology for these microorganisms were published in our previous paper [45]. Seven bacteria were found to precipitate calcium carbonate. The detailed isolation protocol and identification of their calcium carbonate precipitation ability have been reported in the previous study [45].

### Application of bacteria towards precipitation

The three bacteria which produced the highest carbonate precipitation were selected to be used in the sand column experiments described below. The bacteria used were *Pseudomonas nitroreducens* szh_asesj15 (Bacteria isolated from landfill groundwater), *Bacillus* sp. *xjlu_herc15* (Bacteria isolated from leachate), and *Bacillus licheniformis adseedstjo15* (Bacteria isolated from leachate). More information about these bacteria can be found in our previous paper [45]. *Bacillus megaterium* was used as a control in this study, since they outperformed *Sporosarcina pasteurii* at temperatures around 15°C [24,46].

### Biocementation solution and procedure

The cementation solution consisted of 500mM calcium chloride, 500mM urea and bacterial solution (cell density at 2 x 10^7^ cells/ml). Bacterial solution consists of Nutrient broth and bacteria. Nutrient broth consists of Peptone, Yeast extract and sodium chloride. The transport of media through the column occurred due to gravity and diffusion. The experiment was conducted at a temperature of 17°C and atmospheric pressure. Most experiments conducted using porous media have been performed at a temperature >25°C [2,3,7,31,47]. It is well documented that the performance of bacteria to secrete enzymes (e.g. urease, carbonic anhydrase, etc.) is relatively low when the temperature gets lower than 20°C [22]. This study aimed at achieving a more realistic environmental condition for the application of MICP. The cementation solution was replaced 6 times, once every 2 days to allow the cementation to occur. The media was replaced slowly to avoid disturbing the sample content. This became easier as the columns solidified and the permeability decreased. The experiments were carried out without agitation. The indigenous bacteria and control bacteria were tested alongside a blank column. A *control* specimen containing commercially bought *Bacillus megaterium* was used to compare the MICP efficiency of the indigenous bacteria. A blank specimen (without any bacteria) was subjected to the same temperature, pH and the cementation solutions.

### Bacteria optical density

A qualitative calculation of viable biomass optical density (OD) was determined spectrophotometrically at 600 nm (i.e. OD600) using visible light spectrophotometry.

### Column preparation

**Fig 1:**
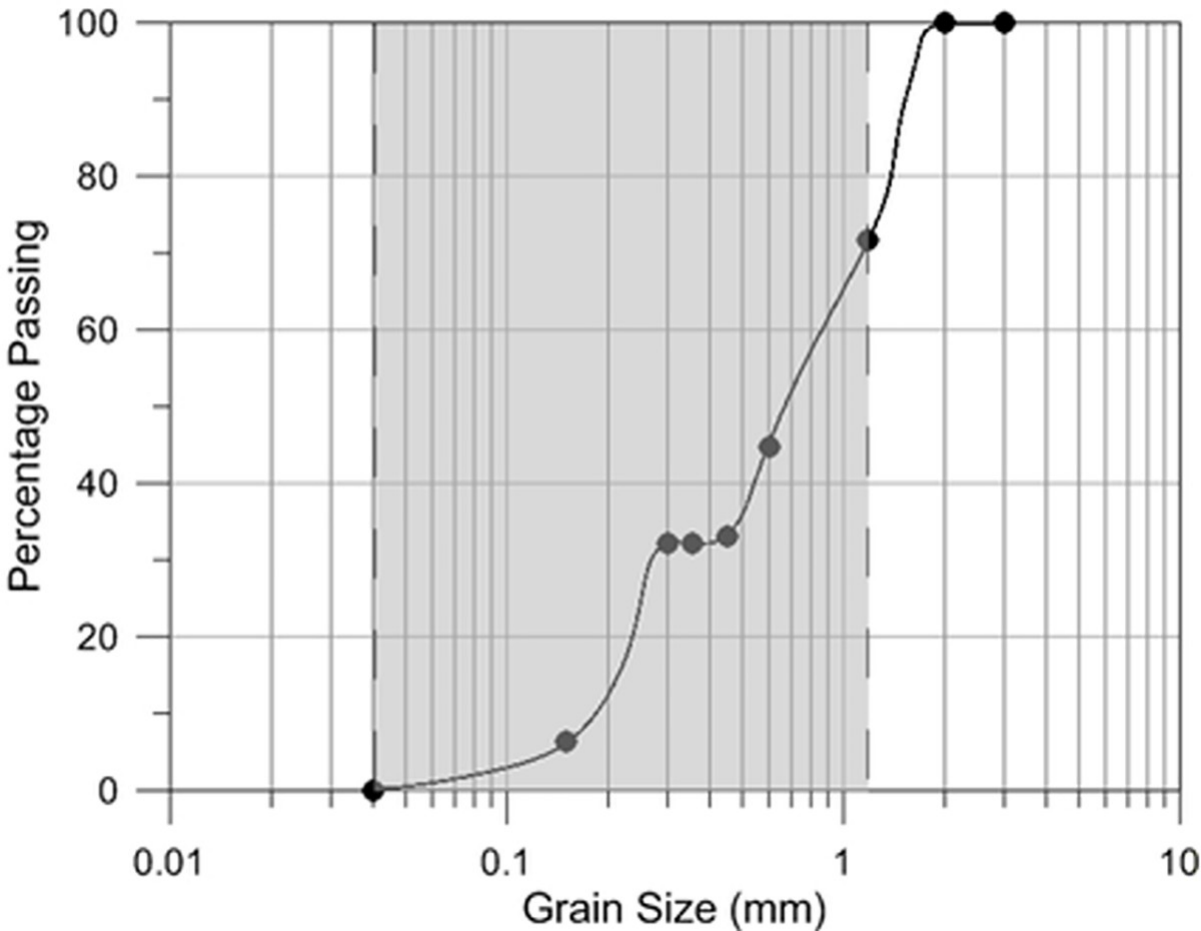
Sand particle size distribution. The size distribution utilised for the experiment is shaded.

**Fig 2:**
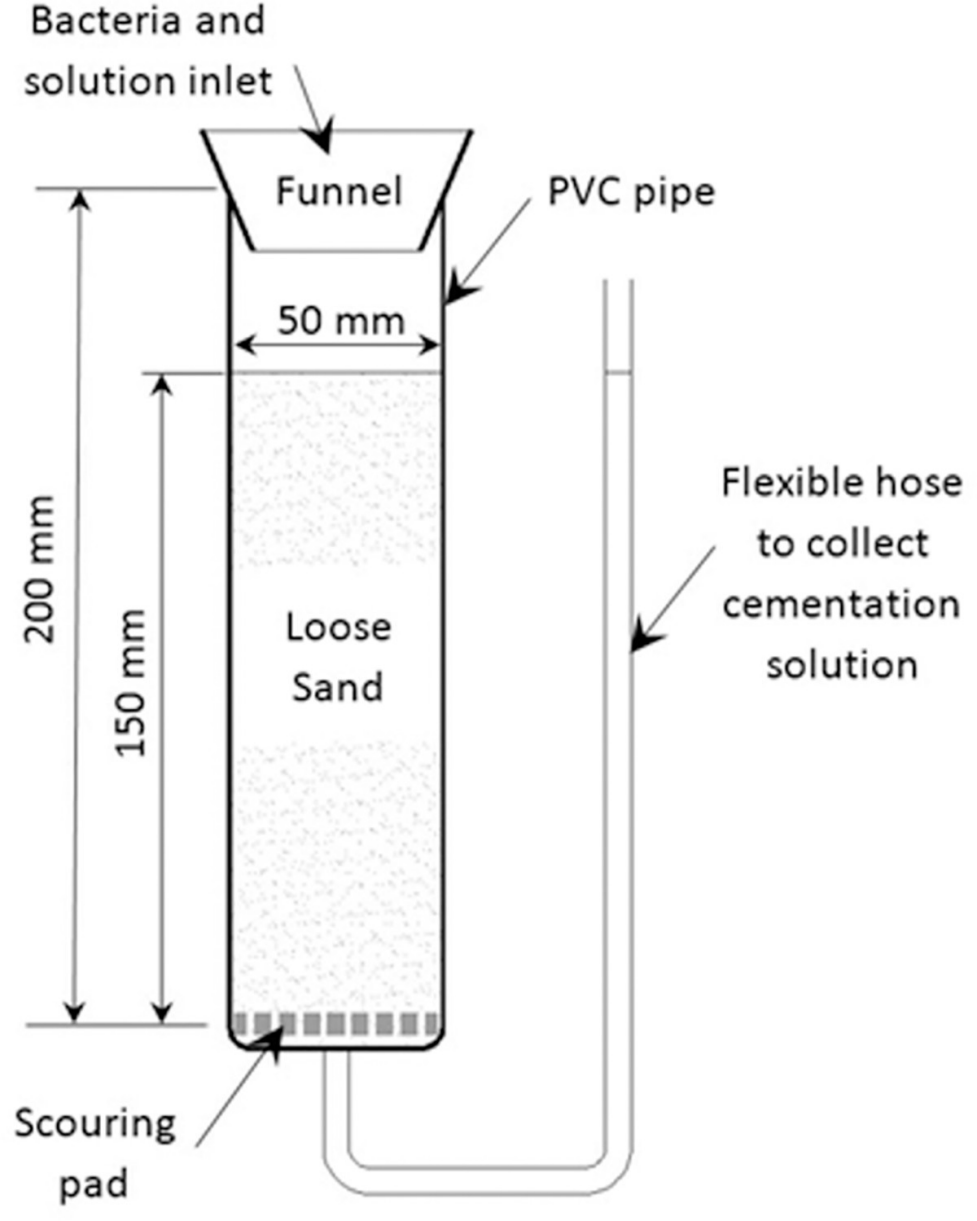
Schematic of the experimental setup of the sand columns.

The soil columns used to evaluate biocementation were modified from a design described in [48]. Pure silica sand (ISO 9000:2001) sieved to between 0.04 and 1.18mm was used for the experiments (Fig 1). Columns were made of Polyvinyl Chloride (PVC) tubing with an internal diameter of 5.5 cm and 20 cm length (Fig 2). The columns were packed with loose dry silica sand. The top part of the column being open to the atmosphere and the bottom part being connected to the inner tube. The media (bacterial suspension and cementation solution) was introduced from the top of the columns. The bottom of the column was covered with a scouring pad layer, to avoid sand loss. A flexible plastic tube of 20mm inner diameter was connected to the outlet of the soil column. In order to maintain cementation solution saturation in the sand column at all times, the outlet pipe was fitted in a u-shape parallel to the column as shown in (Fig 2) [27]. The biocementation media including calcium chloride and urea were filter sterilized. The nutrient broth was autoclaved before adding the bacteria in them. The PVC column, tubes and scouring pad were soaked in 75% ethanol and oven-dried at 70°C.

### Permeability measurement

Estimates of permeability were also made using a falling head procedure [49] between two marks on the inside of the column above the sand. All specimens were also flushed with DI water after finishing the biochemical treatments to remove residual chemicals from the pores throat of coarse sand and then saturated before commencing the permeability test by percolating water through the coarse sand to de-air residual air in the pore matrix. The coefficient of permeability (K) was calculated using the equation:

$$K=2.303\times \left[\frac{aL}{A(t_{f}-t_{i})}\right]\times {log}_{10}\frac{h_{1}}{h_{2}}$$

where *a* is the area of the inlet, L is the distance between the two measuring points, A is the area of the sample, (*t_f_−t_i_*) is the increment of time between two readings, *h*_1_ is head of water above outlet elevation at time *t*_i_ and *h*_2_ head of water above outlet elevation at time *t*_f_.

### Calcium carbonate precipitation

Finally, the extent of precipitation of calcium carbonate was assessed via titration with HCl [50] using approximately 10 grams of randomly subsampled cemented sand from the column. The sand taken for titration was not air-dried as described in Rajasekar et al. (2018). Before this procedure, the sand was washed with DI water to remove excess or unused calcium chloride, urea and any other by-products such as hydrochloric acid that may be retained in sand. The procedure is as follow: weigh the soil sample of 1 to 10 g (±0.001 g) into a 250-mL Erlenmeyer flask: use a volumetric pipette, add 20 mL of standardized 1N HCl to the flask; cover the Erlenmeyer flask with a watch glass and boil the soil-acid mixture for 5 minutes; and add 50–100 mL deionized water using a graduated cylinder. After it has cooled down; add 2 or 3 drops phenolphthalein indicator. Titrate the solution with 1N NaOH solution while swirling the flask and finally record the reading when a faint pink colour develops.

$${CaCO}_{3}equiv.,\%=\left(\frac{V_{HCl}N_{HCl}-V_{NaOH}N_{NaOH}}{grams\;of\;soil}\right)\times 0.05\times 100$$

where V_*HCl*_N_*HCl*_ and V_*NaOH*_N_*NaOH*_ are the volume and normality of HCl and NaOH, respectively.

### Strength measurement

A hand-held penetrometer (PP) was used to measure the penetration resistance of the samples and the readings were converted to unconfined compressive strength (UCS) values to allow comparison with values published elsewhere. This method was mainly selected to ensure continuous monitoring of the strength development. However, only the final strength measurements recorded at 14 days are reported for clarity.

### Qualitative measurement

Scanning electron microscopy (SEM) was used to analyse the presence of calcium carbonate precipitation. Following the physical properties testing, small subsamples of sand agglomerations extracted from the columns, were placed onto pin stubs using double-sided sticky carbon tape and analysed with a scanning electron microscopy (SEM-Hitachi TM3000). The samples were placed uncoated into the microscope to allow a qualitative assessment of the extent of mineral precipitation that occurred after the experiment. The samples were crushed into fine powder and later mounted on a glass slide for the X-ray Diffraction analysis. The samples were tested for their calcium carbonate presence using (Advanced D8, Bruker, Germany).

## Results and discussion

**Fig 3:**
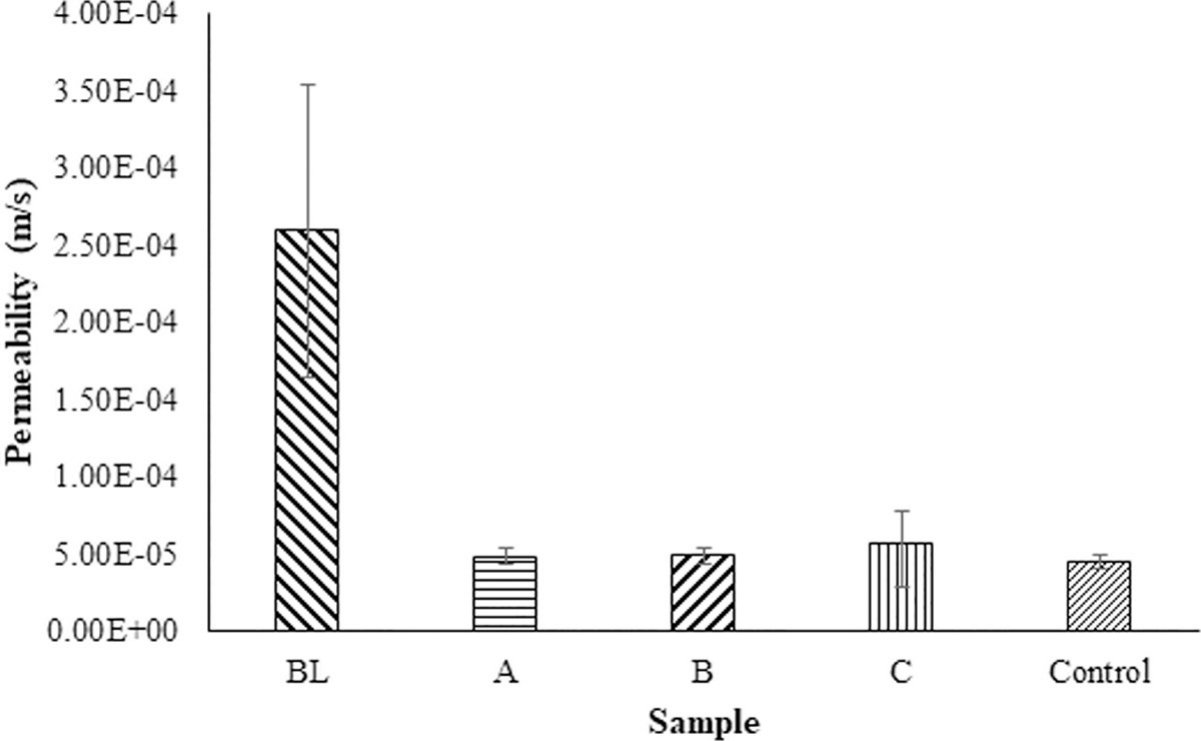
Measurements of soil permeability from the columns containing different biomineralising bacteria. A = *Pseudomonas nitroreducens* szh_asesj15; B = *Bacillus* sp. *xjlu_herc15*; C = *Bacillus licheniformis adseedstjo15* and Control = *Bacillus megaterium*.

In our study, the permeability of the sand columns containing indigenous and commercial bacteria was lower than the control (Fig 3). The mean permeability values produced by the MICP process range from 4.79x10^-5^ ms^-1^ to 5.65x10^-5^ ms^-1^, varying by microbe in comparison to 2.60x10^-4^ ms^-1^ for the control. All the samples had relatively reasonable standard deviations indicative of consistent measurements. The control samples had a more variable flow rate than the other samples varying from 4.83x10^-4^ ms^-1^ to 7.66x10^-4^ ms^-1^. This is thought to be due to variations in cementation (Fig 5).

**Fig 4:**
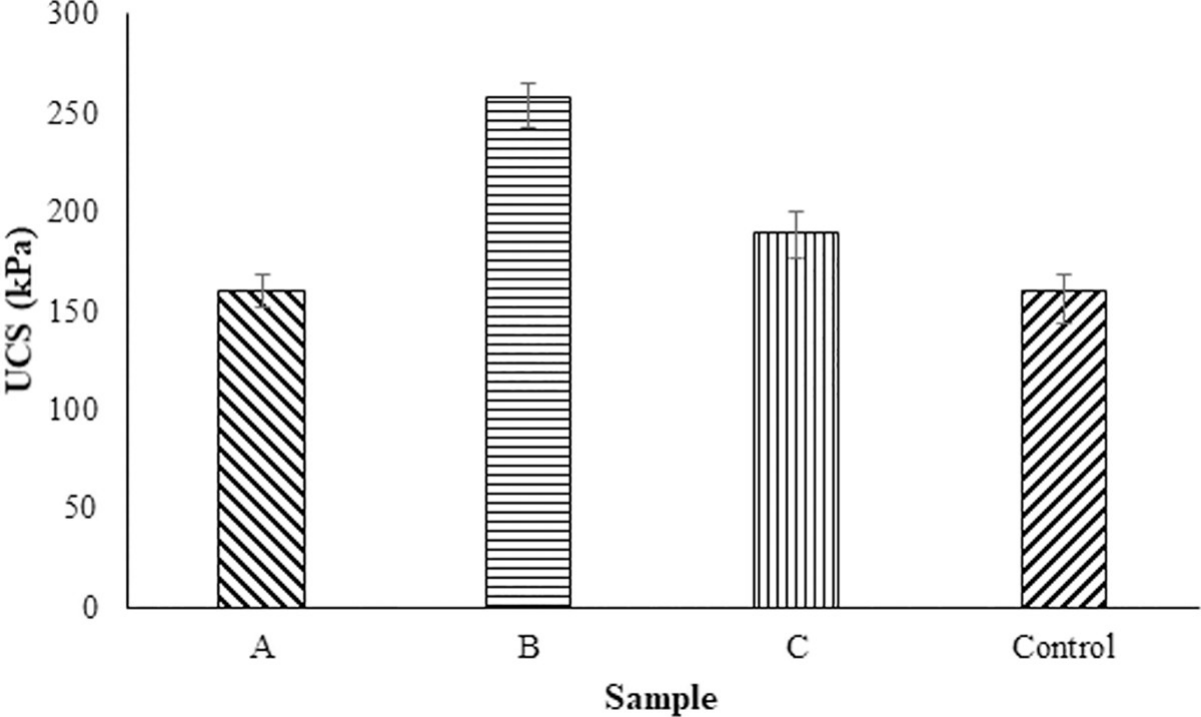
Unconfined compressive strength among indigenous bacteria found in the sand columns. A = *Pseudomonas nitroreducens* szh_asesj15; B = *Bacillus* sp. xjlu_herc15; C = *Bacillus licheniformis* adseedstjo15.

Fig 4 shows the range of unconfined compressive strength measured for the indigenous bacteria and the *control* bacteria. Similar performance can be observed between the samples with Sample B having slightly higher strength than the other samples and the *control* samples. Those values are similar to those produced by others. For example, for MICP utilising the bacteria *Sporosarcina pasteurii*, compressive strengths ranging between 200–240 kPa have been reported where a slow flow treatment approach was used [51], compressive strengths of 290–870 kPa are reported with 42 treatment cycles in a sand column in comparison to the 10 treatment cycles in this study [52]. UCS ranging between 215–932 kPa have been reported where a different organism *Bacillus* sp. VS1 was used however, this was for a 20mm height 10mm width test sample, extracted from a larger structure [2]. A study published by [3] was performed at room temperature (20–25°C) and used twice the concentration of biocementation reagents with a bacteria isolated from a non-contaminated environment. They were able to report higher strength values, in the 0.8 to 1.6 MPa range this could be due to the face that their samples were dried at 60°C before strength measurement. The lack of sand consolidation studies performed at 15°C makes it hard to compare our data with other papers. A study published by [46,53] observed that at 15°C the strength value is higher with *Bacillus megaterium* when compared with *Sporosarcina pasteurii*. In our study, the indigenous bacteria showed higher strength, permeability and CaCO_3_ precipitation than the control bacteria. We believe that the higher performance at lower temperature could be due to the longer retention time of bacteria combined with slow and steady enzyme performance leading to higher urea degradation into carbonate.

**Fig 5:**
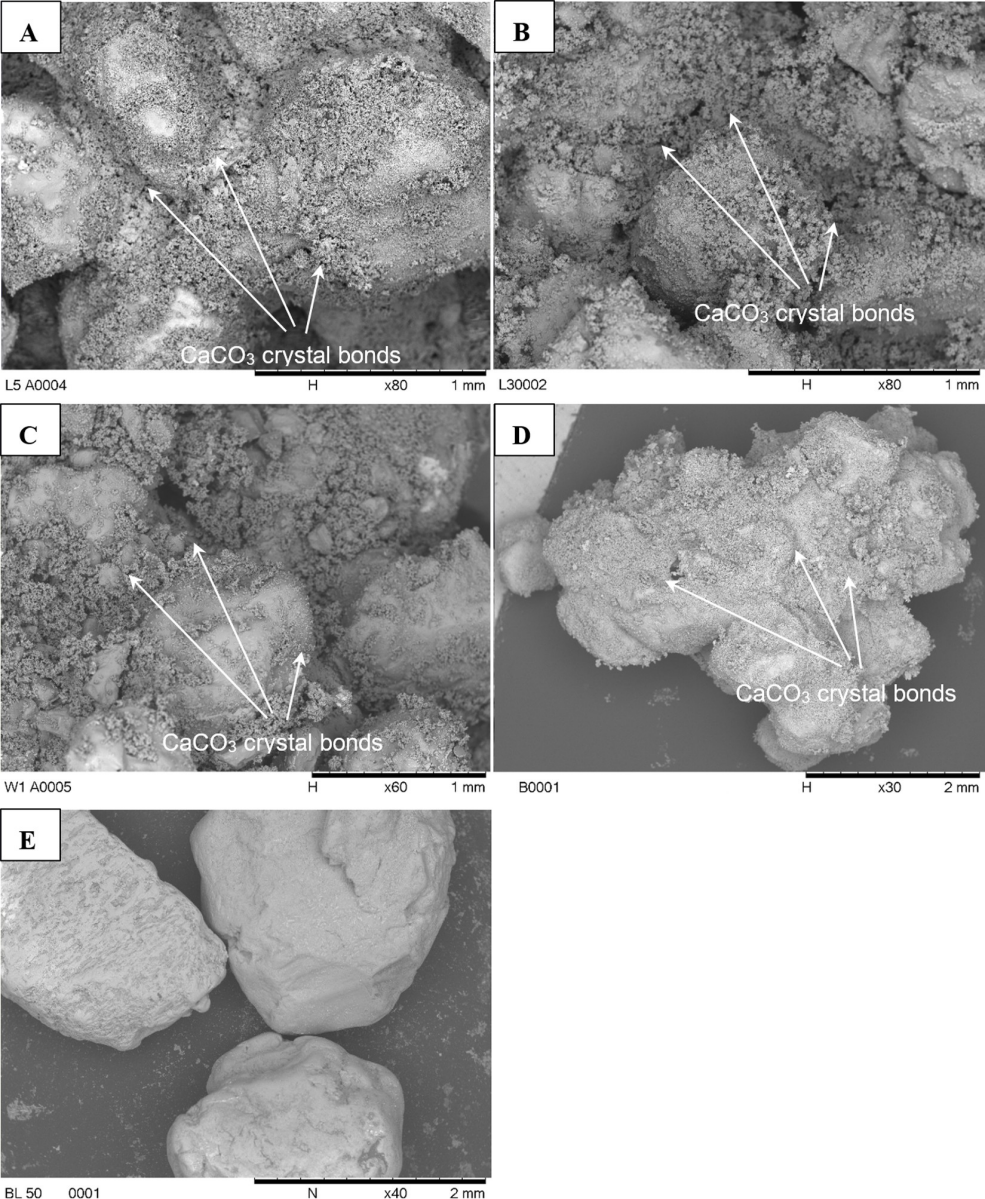
SEM images showing superior cementation between sand particles achieved by the application of MICP in the samples containing indigenous bacteria (A, B and C). Cementation also observed in the *Bacillus megaterium* (control) sample (D). A = *Bacillus licheniformis adseedstjo15*; B = *Bacillus* sp. *xjlu_herc15*; C = *Pseudomonas nitroreducens* szh_asesj15; D = Control (*Bacillus megaterium*) and E = blank (no bacteria).

The growth of crystals caused a reduction in the void ratio of the soils alongside the creation of agglomerated/cemented particles (Fig 5). The reduction in the void ratio resulted in increased strength and decreased permeability in the bacterial columns. When comparing strength and permeability among the bacterial columns, the permeability change was not significantly different compared to strength. This could be attributed to the varied amount of calcium carbonate precipitated among the bacterial columns. The observed morphology of biologically-induced mineral crystals formed varied among organisms (Fig 5). It is generally accepted that the microbe has an impact on the morphology of the crystals, such as those described in Branson, Bonnin [54]. The extracellular precipitation can be associated with the morphologies of the minerals [55]. Hence, the variation in morphology can result from either the interaction of the organism with the crystals, or it can be a secondary effect caused by the extent of the chemical changes in the sand medium generated by the organism [56–58]. An assessment of this can be made by observing the microstructure of the resulting sand columns.

Differences (outlined below) in crystal extent are observed between the biotic and abiotic samples indicating that the rate of growth and extent of the crystals is enhanced by the presence of the microorganisms (Fig 5A–5C). This inferred difference in crystal growth rate is also indicated by the lower pH observed in the control [43,45,59].

As predicted by the permeability tests the observable pore crystal density is approximately equivalent for all the sand samples from the columns containing bacteria. For the control sample (Fig 5D), the crystals appear to be more widely spaced and sparse. There are clusters of crystals in some voids, while others remain entirely free from crystals. The calcite crystals are believed to be affected by factors such as CO_2_ concentration, pH, particle surface charge, rate of carbonation, and reactants [60]. For example, Al Qabany, Soga [61] showed that the precipitation pattern at the pore scale is affected by the injection concentration, and lower chemical concentrations result in better distribution of calcite precipitation. Studies performed by Soon, Lee [62] showed effective calcite crystals formation and bonding employing *B*.*megaterium* concentration of 1 x 10^8^ cfu/mL, cementation reagent concentration of 0.5M, and flow pressure of 1.1bar for a treatment duration of 48 h for residual soil. Higher flow rate (2 bar) resulted in pore-water pressure and disturbance in the media. In addition, the growth of crystals on some grains and not others may be a function of surface roughness/mineralogy.

**Fig 6:**
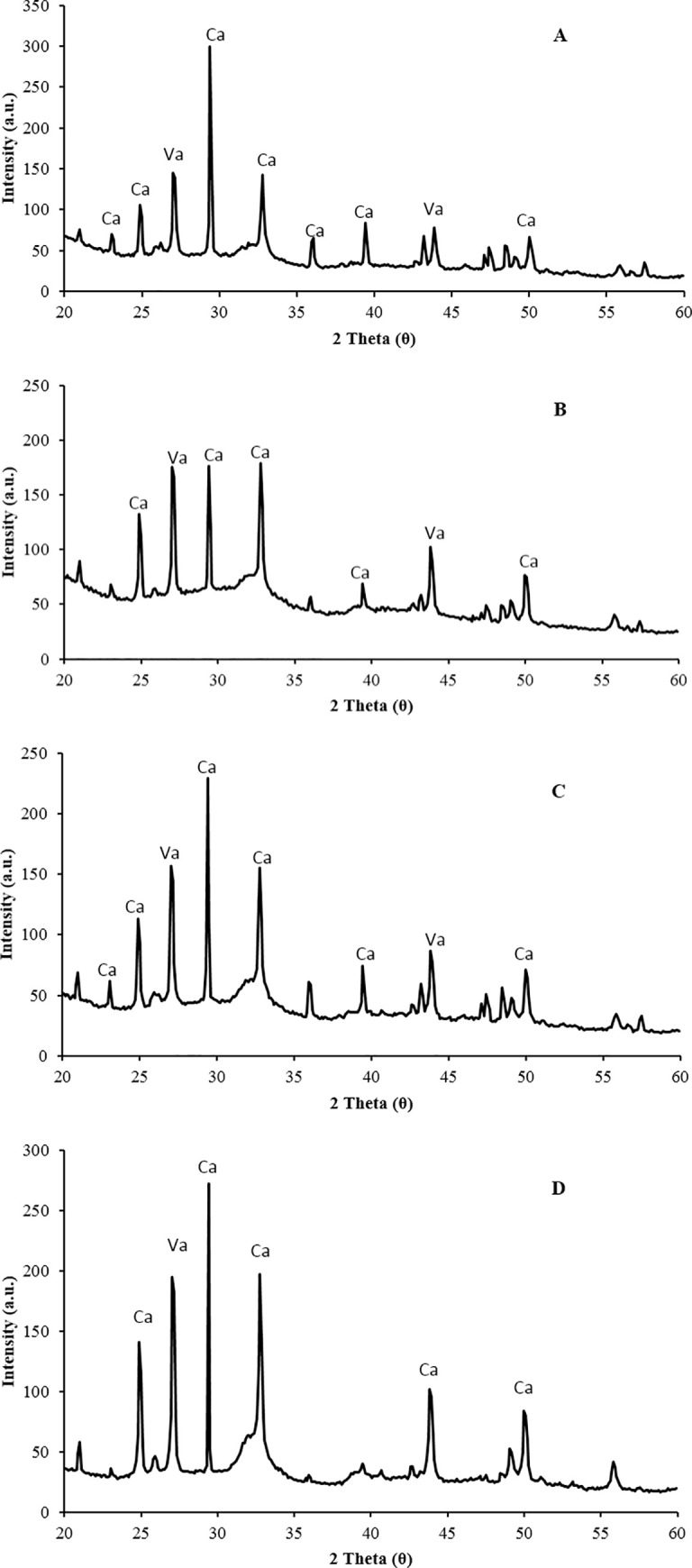
XRD data showing Calcite (Ca) and Vaterite (Va) crystals achieved by the application of MICP in the samples containing indigenous bacteria (A, B and C). A = *Pseudomonas nitroreducens* szh_asesj15; B = *Bacillus* sp. *xjlu_herc15*; C = *Bacillus licheniformis adseedstjo15* and D = Control (*Bacillus megaterium*).

XRD analysis provided information of the composition, and crystalline structure of the biominerals. Calcite and vaterite were precipitated by all the bacterial isolates in this study (Fig 6A–6C). Calcite was found to be dominant in the reference sample when compared to bacterial samples (Fig 6D). This is clearly different from the full spectra for calcite developed in the samples with bacteria. Both vaterite and calcite precipitation are kinetically favoured by the high pH of the fluid phase. Vaterite (which is a metastable phase) can be dissolved and reprecipitated as calcite at low solution supersaturations [63]. They also showed that the rate of dissolution decreases at supersaturation ratios of 1.5 and above. Hence, the presence of urease enzymes may potentially favor calcite formation in the samples and since it is absent in the control, vaterite was found to be dominant. The production of both calcite and vaterite also occurred in other studies; this is particularly the case when calcium chloride is used as a calcium source [6,31,51,64]. Our data also showed vaterite in bacterial samples but at smaller levels which was also reported by several authors [3,6,29,47].

**Fig 7:**
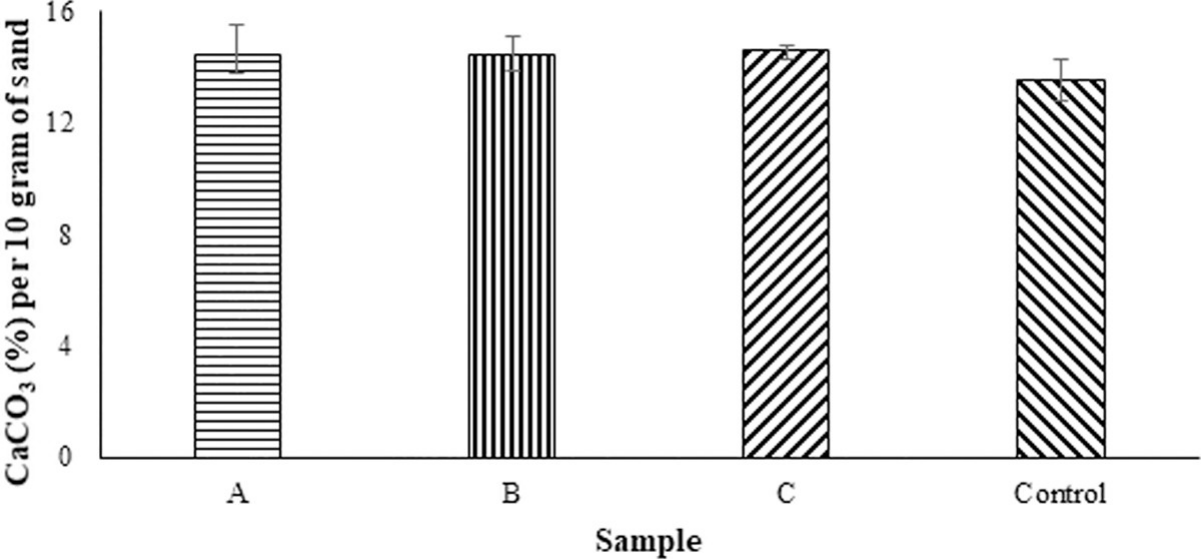
Percentage of CaCO_3_ per 10 grams of sand measured via titration A = *Pseudomonas nitroreducens* szh_asesj15 (Mean: 14.42, Std. Dev.: 0.97); B = *Bacillus* sp. xjlu_herc15 (Mean: 14.63, Std. Dev.: 0.32); C = *Bacillus licheniformis* adseedstjo15 (Mean: 14.43, Std. Dev.: 0.62) and Control = *Bacillus megaterium* (Mean: 13.56, Std. Dev.: 0.72).

The measured percentage of CaCO_3_ in the sand columns containing bacteria measured by titration was higher by approximately 3% compared to that of the *control* sand column (Fig 7). The mean CaCO_3_% produced by the MICP process ranged from 14.42% to 14.63% varying by microbe, compared to 11.75% for the *control* sample. The standard deviations were below 1% for all the samples. Given the measured difference in permeability and strength, a higher difference in CaCO_3_ was expected. It is interesting to note that the difference in CaCO_3_ content between the samples and the control is the result of ~7% void space within the sand media. In an engineering perspective, this difference suggests that the distribution of CaCO_3_ produced by the organism is significant for the modification of permeability and strength.

It is promising that bacteria existing in and thus, resistant to toxic environments have the potential for biomineralization. This may help addressing environmental concerns over the introduction of non-native bacterial species into the environment. Zamarreňo et al. [43] reported that heavy metals concentrations were shown to have an impact on the bacterial diversity within the area from which the bacteria used in this study were isolated. The isolation of MICP capable bacteria from landfill leachate containing high concentrations of heavy metals is indicative of the potential application of this technique in such hazardous conditions. The major challenge to this approach is that it is likely that the presence of MICP capable bacteria may vary and may potentially be absent depending on the location, making it a site-specific technique. In addition, the variation in the strengths that are produced by different micro-organisms may vary creating inconsistencies.

## Conclusions

MICP is currently observed as a promising technique for applications in construction materials and ground improvement. However, there is a need to understand if indigenous bacteria available in contaminated environments such as landfills can precipitate calcium as well as commonly known bacteria. This can open up new possibilities, other than ground improvement, such as heavy metals entrapment avoiding environmental degradation. In that perspective, the indigenous bacteria identified from the landfill and investigated has shown potential to perform better than *Bacillus megaterium*. The permeability reduction of the indigenous bacteria ranged between 4.79 E-05 to 5.6534 E-05 as compared to the control at only 4.56 E-05. This was in line with the amount of CaCO_3_ precipitated, 14.4–14.7% for the indigenous and 13.6% for the control bacteria. In terms of UC strengths, the indigenous bacteria values ranged between 160–258 kPa in comparison to 160 kPa for the control.

In summary, the utilisation of *in situ* organisms within this work was an attempt to assess the existence of potentially MICP microorganisms within the natural engineering environment. Using organisms that are already on site generates much less environmental concern than injecting bacteria which could potentially change the microbial dynamics of the environment. Also, utilizing *in-situ* organisms can potentially reduce the application cost. However, it should also be acknowledged that field application poses several challenges, for e.g., the level of ground improvement produced will be highly dependent on the bacteria present on site and may not be as extensive as that produced using an organism purchased from a culture collection. In addition, the hazardous chemicals present in the leachate may pose too many variables to fully understand the toxicity limits of these organisms.
